# Evaluation of ^89^Zr-DFO Functionalized Manganese–Iron Oxide Core–Shell Nanoparticles as a HER2-Targeted Multimodal Imaging Candidate Nanoplatform

**DOI:** 10.1021/acsomega.6c01561

**Published:** 2026-06-22

**Authors:** Derya Özel, Ayça Tunçel Oral, Selin Güleç, Fatma Yurt

**Affiliations:** † Institute of Nuclear Sciences, Department of Nuclear Applications, 37509 Ege University, Bornova, 35100 Izmir, Turkey; ‡ Medical Imaging Techniques Program, Vocational School of Health Services, Izmir University of Economics, Izmir 35330, Turkey; § Medical Imaging Techniques Program, Cappadocia Vocational College, Cappadocia University, Health Campus, 50420 Nevsehir, Turkey; ∥ The Institute of Natural and Applied Sciences, Department of Biotechnology, Ege University, 35100 Izmir, Turkey

## Abstract

Hybrid multimodal imaging agents combining magnetic resonance imaging (MRI) and positron emission tomography (PET) capabilities offer potential for accurate tumor detection and monitoring. In our previous study, we synthesized manganese-loaded, mesoporous silica-coated superparamagnetic iron oxide nanoparticles functionalized with trastuzumab (Fe_3_O_4_-mSi-NH_2_-Mn-Tra), which demonstrated dual T_1_/T_2_ MRI contrast enhancement and selective cytotoxicity against HER2-positive breast cancer cells in vitro. In the current study, the nanoparticle was labeled with Zirconium-89 (^89^Zr) to evaluate its potential as a HER2-targeted PET imaging agent- candidate. Radiolabeling was performed using the *p*-isothiocyanatobenzyl-desferrioxamine (*p*-NCS-Bz-DFO) chelator, ^89^Zr-DFO-Fe_3_O_4_-mSi-NH_2_-Mn-Tra was labeled of 78 ± 2%. The labeled nanoparticles demonstrated high colloidal stability in PBS for up to 24 h and lipophilic (log *P* = 2.23 ± 0.24). In in vitro uptake studies, radiolabeled nanoparticles showed receptor-specific internalization in HER2-positive SKBR-3 cells (65.25% at 2 h), whereas the uptake was limited in HER2-negative MDA-MB-231 cells (20.22% at 4 h). When the receptor was blocked with trastuzumab, the uptake results was reduced by 11.14 ± 1.2%, confirming HER2-mediated binding. In vivo biodistribution studies in rats have shown that the radiolabeled nanoparticles accumulate in the reticuloendothelial organs (liver 15.53 ± 7.6% ID/g at 120 min) and renal excretion. These results suggest that ^89^Zr-DFO-Fe_3_O_4_-mSi-NH_2_-Mn-Tra nanoparticles may serve as a promising HER2-targeted PET/MRI candidate nanoplatform for further preclinical evaluation.

## Graphical abstract

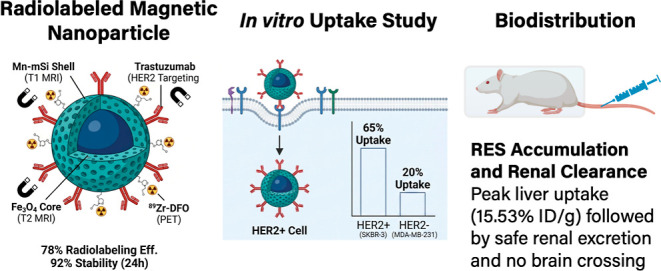

## 1 Introduction

Breast cancer is the most commonly diagnosed malignancy among women worldwide and continues to be one of the leading causes of cancer-related deaths.^1^ This disease exhibits significant molecular heterogeneity, which strongly influences prognosis and response to treatment. Among these, HER2-positive (HER2^+^) tumors, characterized by amplification or overexpression of human epidermal growth factor receptor 2, are approximately 15–20% of breast cancer cases. They exhibit more aggressive clinical behavior and a poorer prognosis.^2,3^ Accurate staging and monitoring of treatment response in HER2^+^ disease requires imaging techniques with high diagnostic accuracy.^4,5^ This need has led to an increasing shift toward multimodal molecular imaging techniques to provide high sensitivity for lesion detection and high anatomical resolution for treatment planning. Molecular imaging methods such as positron emission tomography (PET), single photon emission computed tomography (SPECT), magnetic resonance imaging (MRI), and their hybrid combinations have significantly advanced the noninvasive visualization of tumor biology and microenvironmental changes.^6,7^ Among these, the hybrid positron emission tomography/magnetic resonance imaging (PET/MR) platform, which combines the quantitative capabilities of PET with the superior soft tissue contrast of MRI, has emerged as a leading approach for comprehensive tumor characterization.^8–10^

Despite these advances, a significant need for imaging agents that combine high sensitivity, minimal off-target uptake, suitable pharmacokinetics, and robust translational potential. The development of nanoparticle-based targeted imaging agents offers a promising avenue to overcome these challenges. This is because the tunable size, surface chemistry, and multifunctionality enable the design of probes capable of enhanced tumor accumulation, prolonged circulation, and, crucially, combined imaging and therapeutic (theranostic) capabilities.^11–13^

Superparamagnetic iron oxide nanoparticles (SPIONs) coated with mesoporous silica (mSi) have recently emerged as biocompatible and versatile platforms for multimodal imaging due to their tunable surface functionality, large surface area, and capacity to incorporate additional imaging or therapeutic components.^14^ Incorporation of manganese (Mn) into the silica shell enhances T_1_ relaxation efficiency, while the Fe_3_O_4_ core ensures to T_2_-weighted contrast, thereby enabling dual-mode MRI visualization.^15,16^ Trastuzumab (Tra), a monoclonal antibody targeting HER2, provides receptor-specific binding to HER2-overexpressing tumor cells and converts a passive nanostructure into an active, targeted theranostic agent.^17,18^ In our previous study, Fe_3_O_4_-mSi-NH_2_-Mn-Tra nanoparticles showed excellent dual T_1_/T_2_ MRI contrast and selective cytotoxicity in HER2-positive SKBR-3 cells, confirming their diagnostic selectivity and biological compatibility. In the current study, the nanoparticle was labeled with zirconium-89 (89Zr) via a desferrioxamine (DFO) chelator. The study evaluates its radiolabeling performance, in vitro targeting behavior, and preliminary in vivo biodistribution profile in the context of PET-based assessment.

The development of multifunctional, nanoparticle based probes that combine PET and MR capabilities has attracted significant attention due to the advantages offered by this combination.^19^ Such nanostructures integrate MRI contrast performance with radionuclide labeling, providing simultaneous anatomical, functional, and molecular insights within a single imaging session.^20^ These hybrid imaging platforms enable quantitative whole-body PET tracking alongside high-resolution MRI and may improve lesion detectability, pharmacokinetic assessment, and to therapy-response evaluation.^11,21^

Specificity toward HER2-overexpressing tumors is generally achieved through conjugation of monoclonal antibodies such as trastuzumab to nanoparticle surfaces. This functionalization enhances receptor-mediated endocytosis and selective intracellular accumulation, effectively ensures diagnostic and therapeutic functions within a single construct.^17,18^ Radiolabeled nanoparticles thus offer a unique intersection of nanomedicine and nuclear imaging by combining the radionuclide signal amplification of PET with nanoscale delivery precision, these constructs can overcome sensitivity limitations of conventional imaging probes and provide improved anatomical and functional coregistration through hybrid modalities such as PET/MR or SPECT/CT.^11^ For PET imaging, ^89^Zr is particularly advantageous, as its physical half-life (*t*_1/2_ = 78.4 h) matches the circulation and tumor accumulation kinetics of antibodies and antibody–nanoparticle conjugates^22,23^ The DFO chelator family, particularly *p*-isothiocyanatobenzyl-DFO (DFO-NCS) derivatives, forms kinetically stable complexes with Zr^4+^ under mild labeling conditions, maintaining high radiochemical yield and preserving immunoreactivity. Consequently, DFO-based ^89^Zr labeling has become the clinical gold standard for immuno-PET imaging^24–26^

In this study, we developed and evaluated ^89^Zr-DFO-functionalized Fe_3_O_4_-mSi-NH_2_-Mn nanoparticles conjugated with trastuzumab (^89^Zr-DFO-Fe_3_O_4_-mSi-NH_2_-Mn-Tra). Building on our prior MRI-based findings, which demonstrated dual T_1_/T_2_ contrast enhancement and HER2 specificity, we now focus on assessing the radiochemical labeling efficiency, stability, lipophilicity, receptor-mediated cellular uptake, and in vivo biodistribution of this novel hybrid construct. Collectively, these results are expected to elucidate the interplay between physicochemical properties and biological performance in HER2-targeted hybrid nanoprobes and to provide preliminary insight into their potential as PET/MR candidate platforms for breast cancer imaging.

## 2 Materials and Methods

### 2.1 Materials

All reagents and chemicals were of analytical grade. Zirconium-89 (^89^Zr) in the form of ^89^Zr-oxalate (1 M oxalic acid) was purchased from the Eczacıbaşı Monrol Radiochemistry Nuclear Products, Turkiye. Trastuzumab (Herceptin) was used as the targeting ligand. The preparation of the core–shell Fe_3_O_4_-mSi-NH_2_-Mn-Trastuzumab (Tra) nanoparticles had been previously reported in our study.^27^ The most commonly used chelating agent *p*-NCS-Bz-DFO­[N1-hydroxy-N1-(5-(4-(hydroxy­(5-(3-(4-isothiocyanatophenyl) thioureido)­pentyl)­amino)-4-oxobutanamido)­pentyl)-N4-(5-(*N*-hydroxyacetamido)­pentyl) succinamide (*p*-isocyanato-benzyl-desferrioxamine)] was obtained from Macrocyclics (Dallas, USA).

### 2.2 Preparation of *p*-NCS-Bz-Deferoxamine (DFO) Fe_3_O_4_-mSi-NH_2_-Mn-Tra Magnetic Nanoparticles

The bifunctional chelator *p*-isothiocyanatobenzyl-desferrioxamine (DFO-NCS) was covalently coupled to the surface amine groups of Trastuzumab-functionalized magnetic nanoparticles.^23,28,29^ Fe_3_O_4_-mSiO_2_-NH_2_-Mn-Tra nanoparticles (3.5 mg) were dispersed in 3 mL phosphate-buffered saline (PBS, pH 7.4). Then, we adjusted the pH to 8.5 using 0.1 M NaHCO_3_ (pH 9.0) to promote nucleophilic substitution between the −NH_2_ and −NCS moieties. A 20 mg/mL DFO-NCS stock solution was freshly prepared in dimethyl sulfoxide (DMSO), and 30 μL of the solution (approximately 600 μg) was added to the nanoparticle suspension. The reaction was carried out at 37 °C with orbital shaking agitation (600 rpm) for 2 h in the dark. Following incubation, DFO-conjugated nanoparticles were collected by centrifugation at 10,000 rpm for 30 min at 4 °C and washed three times with 0.5 M HEPES buffer (pH 7.0) to remove unreacted chelator. The purified DFO-Fe_3_O_4_-mSiO_2_-NH_2_-Mn-Tra nanoparticles were lyophilized and stored at −20 °C until radiolabeling. The conjugation occurred via the formation of a stable thiourea linkage between the isothiocyanate group of DFO and surface amines, consistent with previously reported DFO-based chelation strategies.^23,26,30,31^

### 2.3 Preparation of *p*-NCS-Bz-Deferoxamine (DFO) Chelating Agent-Bound to Trastuzumab Antibody

The chelator *p*-isothiocyanatobenzyl-desferrioxamine (DFO-NCS) was conjugated to Trastuzumab through covalent coupling between the chelator’s isothiocyanate group and the antibody’s surface amines.^32,33^ Covalent attachment through the thiourea bond was similar to previously reported DFO-based immunoconjugation protocols.^23,31,33^ Trastuzumab (1 mg) was dissolved in 3 mL phosphate-buffered saline (PBS, pH 7.4), and the pH was adjusted to 8.5 using 0.1 M sodium bicarbonate to facilitate nucleophilic substitution. A 30 μL (600 μg) of DFO-NCS from prepared a 20 mg/mL stock solution was added to the Trastuzumab solution. The reaction mixture was stirred at 37 °C and 600 rpm for 2 h in the dark. Unreacted DFO was removed by centrifugal ultrafiltration using Amicon Ultra-50 kDa (MWCO) centrifugal filter units at 10,000 rpm for 30 min at 4 °C. Upon completion of the conjugation, the retained conjugate was washed three times with 0.5 M HEPES buffer (pH 7.0) to ensure complete removal of unbound chelator. The purified DFO-Tra conjugate was immediately used for radiolabeling experiments on the same day.

### 2.4 Characterization Studies of DFO-Fe_3_O_4_-mSi-NH_2_-Mn-Tra Nanoparticles and DFO-Tra

The hydrodynamic size and zeta potential of the DFO-Fe_3_O_4_-mSi-NH_2_-Mn-Tra nanoparticles and Fe_3_O_4_-mSi-NH_2_-Mn-Tra conjugate were measured using dynamic light scattering (DLS), on a Malvern Zetasizer Nano ZS equipped with a fixed scattering angle of 173° (backscatter detection). All measurements were performed at 25 °C in zeta cuvettes. Each sample was measured in triplicate (*n* = 3), and the results are reported as the mean ± standard deviation (SD). The polydispersity index (PDI) was simultaneously recorded to assess the colloidal homogeneity of each formulation. The samples obtained after lyophilization were dispersed in ultrapure water using a homogenizer. The analysis was performed to evaluate and confirm the structural integrity and surface charge characteristics of the nanoparticle system. Fourier Transform Infrared Spectroscopy (FTIR) analysis (PerkinElmer Spectrum) was performed to demonstrate the formation of bonds between the nanoparticle surface and DFO, and between DFO and the antibody, as well as to confirm the structural characterization.

### 2.5 Radiolabeling with Zirconium-89

^89^Zr-oxalate was neutralized using 1 M Na_2_CO_3_. 500 μg DFO-Fe_3_O_4_-mSi-NH_2_-Mn-Tra or DFO-Tra was dissolved in 0.5 mL of HEPES buffer (0.5 M, pH 7.0) and added 500 μCi ^89^Zr-oxalate solution. The mixture was incubated at 37 °C for 30 min agitation at 600 rpm.^31^ Radiolabeling efficiency and purity were determined by thin-layer radiochromatography.

#### 2.5.1 Quality Control of Radiolabeled Compounds

The radiolabeling efficiency of both ^89^Zr-DFO-Fe_3_O_4_-mSi-NH_2_-Mn-Tra and ^89^Zr-DFO-Tra complexes was determined using thin layer radiochromatography (TLRC), as previously described.^31,34^ Merck Cellulose F strips (1 × 10 cm) were used as the stationary phase. After completion of the radiolabeling reaction, 2–3 μL of the labeled samples (0.1–0.2 MBq) were spotted approximately 1 cm from bottom of each strip. All TLRC analyses were performed in triplicate (*n* = 3), and radiolabeling efficiencies are reported as mean ± SD. Radiochemical purity was evaluated using three different mobile phases: phosphate-buffered saline (PBS, pH 7.4); 100 mM diethylenetriaminepentaacetic acid (DTPA, pH 5.0); and 20 mM citric acid (pH 5.0) The strips were developed to approximately 9 cm solvent front migration in each mobile phase using a standard ITLC development chamber. After development, they were removed, air-dried at room temperature, and analyzed using a Bioscan AR-2000 radio-TLC scanner (Bioscan, USA). The radiochromatograms were evaluated to determine Rf values and radiolabeling efficiencies.

#### 2.5.2 In Vitro Stability Assay

The in vitro stability of ^89^Zr-DFO-Fe_3_O_4_-mSi-NH_2_-Mn-Tra and ^89^Zr-DFO-Tra was evaluated over 24 h in PBS (pH 7.4) at 37 °C. Following the optimized radiolabeling procedure, 100 μL of each radiolabeled compound (0.1–0.2 MBq) was added into 500 μL PBS (pH 7.4) in microtubes and incubated at 37 °C. The sample were collected at 0.5, 1, 2, 4, and 24 h for radiolabeled compounds. At each time point, radiochemical purity was determined by TLRC method. The radiochemical stability of the labeled constructs over 24 h was evaluated by linear regression analysis of radiochemical purity versus time.

#### 2.5.3 Determination of Lipophilicity (log *P*)

Lipophilicity was assessed using the *n*-octanol/water partition method.^35^ 100 μL of the radiolabeled compounds (0.1–0.2 MBq) was added with 3.0 mL *n*-octanol and 3.0 mL deionized water in glass vials pre-equilibrated at room temperature. The biphasic mixtures were vortexed for 1 h to phase distribution and subsequently centrifuged at 3000 rpm for 5 min for each phase separation. The samples (500 μL) was taken from both the aqueous and organic phase and counted in a gamma counter. The lipophilicity of the compounds was determined from the activity of the *n*-octanol/water ratio (log *P*). Each experiment was performed in three independent replicates, and results were reported as mean log *P* ± SD.

### 2.6 In Vitro Cell Uptake Assay

The cellular uptake of ^89^Zr-DFO-Fe_3_O_4_-mSi-NH_2_-Mn-Tra and ^89^Zr-DFO-Tra was evaluated using two human breast cancer cell lines with HER2 expression profiles. SKBR-3 cells are established as a HER2-overexpressing (HER2^+^) cell line, while MDA-MB-231 cells serve as a HER2-negative control. The HER2-dependent differential biological behavior of these cell lines toward the same nanoplatform was functionally validated in our preceding study,^27^ in which trastuzumab-conjugated nanoparticles demonstrated HER2-selective cytotoxicity (IC_50_: 3.12 μg/mL in SKBR-3 vs 6.25 μg/mL in MDA-MB-231), enhanced apoptosis induction in SKBR-3 cells by flow cytometric Annexin V analysis, and preferential dual-mode T1/T2MRI contrast enhancement in the HER2-positive cell line. Cells were maintained in Dulbecco’s Modified Eagle Medium (DMEM) supplemented with 10% fetal bovine serum (FBS), 1% l-glutamine, 1% nonessential amino acids (NEAA), and 1% sodium pyruvate at 37 °C in a humidified atmosphere containing 5% CO_2_ incubator.

At confluence, cells were seeded into 12-well plates at a density of 5 × 10^5^ cells per well and incubated for 24 h to allow for adhesion. After incubation, each well was washed with 500 μL of fresh culture medium to remove nonadherent cells. Subsequently, 450 μL of complete medium and 50 μL of the ^89^Zr-labeled compounds were added to each well, yielding a total activity of 0.1 MBq per well. The initial activity (*A*_0_) of each well was measured using a gamma counter. The radiolabeled formulations were incubated with SKBR-3 and MDA-MB-231 breast cancer cells for 1, 2, 4, and 24 h. At each time point, wells were washed twice with 500 μL cold PBS to remove unbound or surface-associated radioactivity. After washing, the remaining activity (*A*_1_) associated with the cells was measured using a gamma counter. The cell uptake radioactivity was quantified. The percentage of cellular uptake was calculated as $\%\;Uptake=\left(\frac{A_{1}}{A_{0}}\right)\times 100$ . This methodology allowed for direct quantitative comparison of nanoparticle-mediated and antibody-mediated radiotracer internalization in HER2-positive and HER2-negative cell lines.^26,33^ Statistical analyses were performed using GraphPad Prism software.

### 2.7 Receptor Blocking (Competition) Assay

A receptor blocking assay was performed to confirm the HER2-specific binding of ^89^Zr-DFO-Fe_3_O_4_-mSi-NH_2_-Mn-Tra and ^89^Zr-DFO-Tra. The assay was conducted using SKBR-3 (HER2^+^) and MDA-MB-231 (HER2^–^) breast cancer cell lines under identical culture conditions as described in the uptake studies.

Cells were seeded into 12-well plates at a density of 5 × 10^5^ cells per well and incubated for 24 h at 37 °C, 5% CO_2_. The seeded wells were divided into two groups (Trastuzumab saturated and nonsaturated groups). For blocking conditions, the culture medium in each well was replaced with 500 μL of complete DMEM containing an excess of unlabeled trastuzumab (500 nM). Cells were preincubated with the antibody for 1 h, allowing receptor saturation. After the incubation period, the supernatant was removed, and each well was washed with 500 μL of medium. Then, 500 μL of Trastuzumab was added to the specified wells for saturation and incubated for 1 h. Postincubation, the medium was removed, and each well was washed with 500 μL of cold PBS.

Following preincubation, 50 μL of radiolabeled compound (^89^Zr-DFO-Fe_3_O_4_-mSi-NH_2_-Mn-Tra or ^89^Zr-DFO-Tra, 0.1 MBq per well) was added to the blocking wells. Parallel wells without cold antibody served as unblocked controls. The initial activity (*A*_0_) in each well was measured using a gamma counter as soon as the radiolabeled compound was added.

Cells were incubated with the radiotracers for 1, 2, 4, and 24 h. At each time point, wells were washed twice with 500 μL cold PBS to remove unbound activity. The remaining cell-associated activity (*A*_1_) was counted using a gamma counter.The percentage of radiotracer uptake under blocked and unblocked conditions was calculated using

$$\%\;Uptake=\left(\frac{A_{1}}{A_{0}}\right)\times 100$$

### 2.8 Biodistribution of ^89^Zr-DFO-Fe_3_O_4_-mSi-Mn-Tra Nanoparticles

The in vivo biodistribution of ^89^Zr-DFO-Fe_3_O_4_-mSi-NH_2_-Mn-Tra was evaluated in a rat model to assess systemic distribution and organ-level accumulation. All animal experiments were approved by the Ege University Local Ethics Committee for Animal Experiments (HAYDEK) under protocol number 2024-102, and were conducted in accordance with the European Directive 2010/63/EU on the protection of animals used for scientific purposes and the ARRIVE 2.0 guidelines. Female Wistar rats (6–8 weeks old, 220–250 g) were housed under standard laboratory conditions (12 h light/dark cycle, 22 ± 2 °C, 55 ± 10% relative humidity) with free access to food and water. Animals were randomly assigned to four experimental groups corresponding to different sacrifice time points 60, 120, 240, and 1440 min postinjection; *n* = 3 per group. Prior to administration, the nanoparticle formulation was radiolabeled with ^89^Zr, and radiochemical purity (>95%) was confirmed by Instant Thin-Layer Radio Chromatography (ITLRC).

A dose of 150 μCi (5.55 MBq) of ^89^Zr-DFO-Fe_3_O_4_-mSi-Mn-Tra (in 200 μL of sterile PBS) was injected to the each rat intravenously into the tail vein. At the designated time points postinjection, animals were sacrificed, and the major organs and tissues (blood, liver, spleen, kidneys, heart, stomach, intestines, brain, breast, ovary, and uterus) were removed through systematic dissection and weighed using a microbalance to obtain wet tissue mass.

Tissue-associated radioactivity was quantified using a gamma counter. All measurements were corrected for background and physical decay to the time of injection. For each organ, the percentage of injected dose per gram of tissue (% ID/g) was calculated according to

$$\%\;ID/g=\frac{A_{organ}}{A_{injected}\times m_{organ}}\times 100$$

where *A*_organ_ is the activity measured in the organ (MBq), *A*_injected_ is the total injected activity (MBq), and *m*_organ_ is the tissue weight (g). The biodistribution data (% ID/g) were summarized as measured ± standard deviation (SD) for each organ and time point. These data were then utilized to construct time–activity profiles, which describe systemic distribution, clearance kinetics, and reticuloendothelial system (RES) uptake of ^89^Zr-DFO-Fe_3_O_4_-mSi-Mn-Tra. were summarized as measured ± standard deviation (SD) for each organ and time point and used to construct time–activity profiles describing systemic distribution, clearance kinetics, and reticuloendothelial system (RES) uptake of ^89^Zr-DFO-Fe_3_O_4_-mSi-Mn-Tra.

### 2.9 Statistical Analysis

All quantitative data are expressed as mean ± standard deviation (SD) from three independent replicates (*n* = 3) unless otherwise stated. All statistical analyses were conducted using Graphpad Prism 8.4.2. software.

## 3 Results and Discussion

### 3.1 Physicochemical Characterization of DFO-Conjugated Nanoparticles

#### 3.1.1 Hydrodynamic Size and Zeta Potential Analysis

Dynamic light scattering (DLS) measurements revealed that conjugation of DFO to the Fe_3_O_4_-mSi-NH_2_-Mn-Tra nanoparticles resulted in an increase in hydrodynamic diameter (Z-average) from 246.2 ± 12.8 nm to 294.7 ± 0.4 (Table 1). DFO conjugation resulted in a statistically significant increase in hydrodynamic diameter (from 246.2 ± 12.8 to 294.7 ± 0.4 nm; *p* = 0.003), corresponding to a 48.4 nm increase (19.7%). Rounding has been standardized throughout, and the table footnote now specifies the error type (SD), replicate number (*n* = 3), and statistical test used. This size enlargement is consistent with the successful surface functionalization of the nanoparticle by the bulky DFO ligand, which introduces additional surface moieties and hydration layers. Similar increases of 10–20% in hydrodynamic diameter following DFO conjugation have been reported for other DFO–nanoparticle systems.^36,37^

**Table 1:** Size and Zeta Potentials of Nanoparticles

nanoparticles	Size (nm)	PDI	zeta potential (mV)
Fe_3_O_4_-mSi-NH_2_-Mn-Tra	246.2 ± 12.8	0.195 ± 0.122	–18.7 ± 5.1
DFO-Fe_3_O_4_-mSi-NH_2_-Mn-Tra	294.7 ± 0.4	0.367 ± 0.075	–16.0 ± 1.0

The polydispersity index (PDI) of Fe_3_O_4_-mSi-NH_2_-Mn-Tra was 0.195 ± 0.122, indicating a narrow size distribution. Following DFO conjugation, the PDI increased to 0.367 ± 0.075, reflecting moderate polydispersity consistent with heterogeneous covalent surface functionalization, in which the DFO chelator attaches to surface amine groups with varying accessibility across the nanoparticle population (Table 1).

The zeta potential changed from −18.7 ± 5.1 mV to −16.0 ± 1.0 mV, confirming that the nanoparticles retained an overall negative surface charge, favorable for colloidal stability under physiological pH. The slight shift toward neutrality can be attributed to the introduction of neutral thiourea and hydroxamate groups of DFO onto the aminated silica surface.^37,38^ DLS size distribution histograms and zeta potential profiles are presented in Supplementary Figure S1 These findings indicate that DFO functionalization did not affect the colloidal integrity of the nanoparticles.

#### 3.1.2 FTIR Spectroscopy Characterization

FTIR spectroscopy was employed to confirm the successful conjugation of DFO to the nanoparticle (Figure 1) and trastuzumab components (Figure 2). In the spectrum of the parent Fe_3_O_4_-mSi-NH_2_-Mn-Tra nanoparticles, pronounced absorption bands were observed at 1050–1100 cm^–1^, corresponding to the stretching vibrations of Si–O–Si groups, and at 550–600 cm^–1^, which were assigned to Fe–O lattice vibrations. Following the DFO functionalization process, the presence of additional bands were detected at 2858–2931 cm^–1^ and 2995–3004 cm^–1^, which were attributed to the asymmetric and symmetric stretching modes of −CH_2_ and −NH groups, respectively. Importantly, distinct absorptions were identified at 1566 cm^–1^ (amide II, N–H bending), 1628 cm^–1^ (amide I, C=O stretching), and a shoulder near 1600 cm^–1^, corresponding to the C=N stretching of the thiourea linkage. The presence of these bands was interpreted as direct evidence that DFO was covalently bound to the amine-functionalized nanoparticle surface via its isothiocyanate group.^35,39^ For the DFO–trastuzumab conjugate, the characteristic protein amide I and II bands at 1650–1550 cm^–1^ were retained together with the DFO-specific C=O and N–O absorptions. The preservation of these features indicated that the structural integrity of both trastuzumab and DFO was maintained during conjugation which is a critical requirement for preserving immunoreactivity.^23^

**Figure 1:**
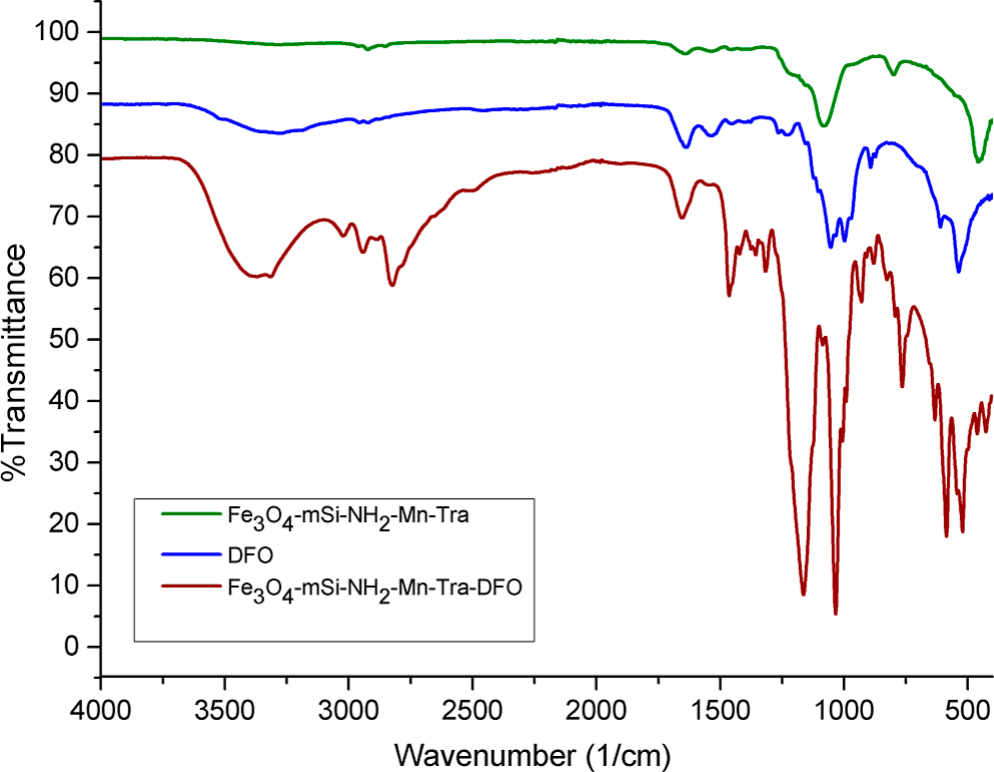
FTIR spectrum of Fe_3_O_4_-mSi-NH_2_-Mn-Tra and DFO conjugated Fe_3_O_4_-mSi-NH_2_-Mn-Tra nanoparticles and DFO.

**Figure 2:**
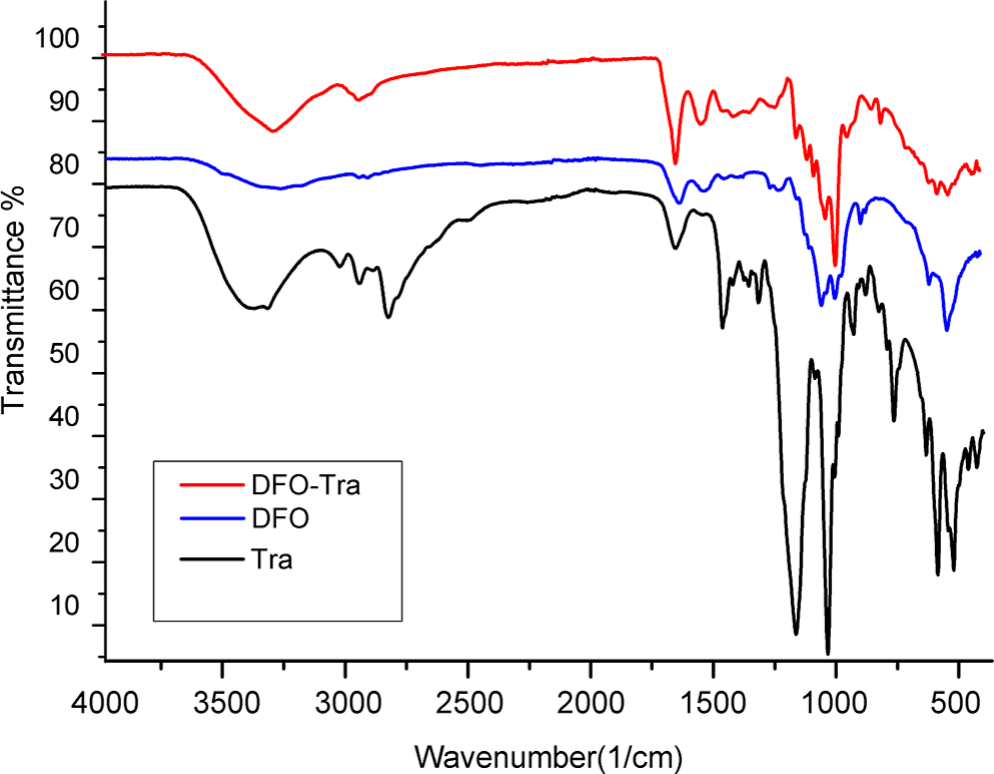
FTIR spectrum of Tra, DFO and DFO conjugated Tra.

In result, the FTIR spectra demonstrated strong spectroscopic evidence that DFO had been successfully conjugated to both the nanoparticle and antibody without structural degradation. This finding is significant for radiolabeling compatibility and biological robustness.

### 3.2 Radiolabeled Samples Quality Control Results

Radiolabeling of the DFO-functionalized magnetic nanoconjugate with zirconium-89 was successfully achieved under optimized conditions, and labeling efficiency were assessed via thin-layer radiochromatography (TLRC). Radiolabeled samples were spotted on TLRC plates and developed in three mobile phases: A (PBS, pH 7.4), B (100 mM DTPA, pH 5), and C (20 mM citric acid, pH 5). As summarized in Table 2, free ^89^Zr migrated in all solvent systems (*R*_*f*_ ≈ 0.85–0.99) (Figure 3), while ^89^Zr-DFO-Fe_3_O_4_-mSi-NH_2_-Mn-Tra (Figure 4) and ^89^Zr-DFO-Tra (Figure 5) remained at the origin when used PBS and DTPA mobil phases, thereby confirming efficient chelation and the absence of unbound radionuclide species.^23,28^ Notably, in citric acid mobile phase (pH 5.0), ^89^Zr-DFO-Fe_3_O_4_-mSi-NH_2_-Mn-Tra exhibited an *R*_*f*_ value of 0.93 ± 0.03, which is comparable to that of free ^89^Zr (*R*_*f*_ = 0.98), indicating substantial migration of the radiolabel toward the solvent front. This behavior was not observed for the antibody conjugate ^89^Zr-DFO-Tra (*R*_*f*_ value 0.09 ± 0.02) in the same mobil phases. The high *R*_*f*_ value observed for the nanoparticle in citric acid is attributed to the competitive transchelation of Zr^4+^ by citrate ions at low pH, which can displace the metal from the DFO chelator on the nanoparticle surface. The nanoparticle’s large surface area and the partial exposure of DFO chelators at the particle–solvent interface may render the chelate more susceptible to acid-mediated dissociation compared with the antibody conjugate, where DFO is more intimately associated with the protein scaffold. Importantly, when developed in PBS (pH 7.4) and DTPA (pH 5.0), the nanoconjugate remained at the origin (*R*_*f*_ = 0.06 and 0.25, respectively), confirming stable chelation under conditions more representative of the physiological environment. The radiolabeling efficiency of nanocomposites was evaluated, two temperature conditions (room temperature and 37 °C). Incubation times of 0.5, 1, 2, and 4 h were evaluated, and the optimal radiolabeling conditions were identified as 37 °C, pH 7.0–7.5, and 30 min (Table 3). Under these conditions, the radiolabeling efficiencies were 78 ± 2% for ^89^Zr-DFO-Fe_3_O_4_-mSi-NH_2_-Mn-Tra and 98 ± 2% for ^89^Zr-DFO-Tra (Figures 4 and 5). These results demonstrated that Zirconium-89 was effectively coordinated to the DFO chelating groups without compromising the nanoparticle’s colloidal stability. The yields are consistent with previously reported DFO-mediated ^89^Zr radiolabeling of antibody and nanoconjugate systems, typically ranging between 70–95%.^26,33^ Consequently, these data confirmed that Zirconium-89 can be incorporated into multifunctional nanoparticles, maintaining both radiochemical integrity and biological compatibility.

**Table 2:** *R*_f_ Values^a^

	A (PBS, pH:7.4)	B (100 mM DTPA)	C (20 mM citric acid)
89Zr	0.99 ± 0.07	0.85 ± 0.05	0.98 ± 0.03
89Zr-DFO-Tra	0.1 ± 0.01	0.05 ± 0.01	0.09 ± 0.02
89Zr-DFO-Fe3O4-mSi-NH2-Mn-Tra	0.06 ± 0.01	0.25 ± 0.1	0.93 ± 0.03

a Data represent mean ± SD (*n* = 3).

**Figure 3:**
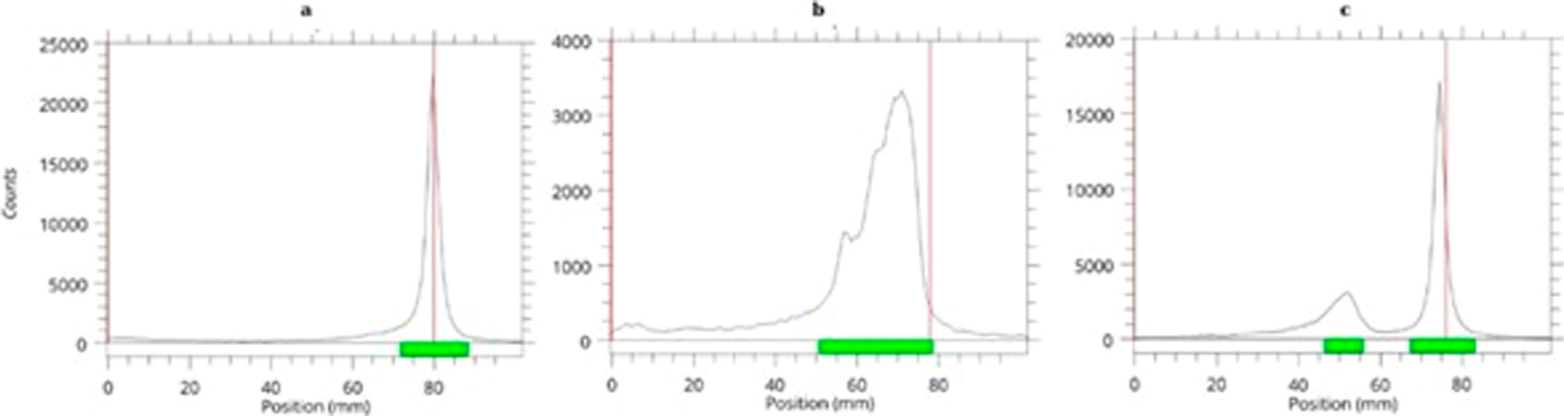
TLRC chromatograms of ^89^Zr oxalate in (a) PBS, (b) DTPA, and (c) citric acid mobil phases.

**Figure 4:**
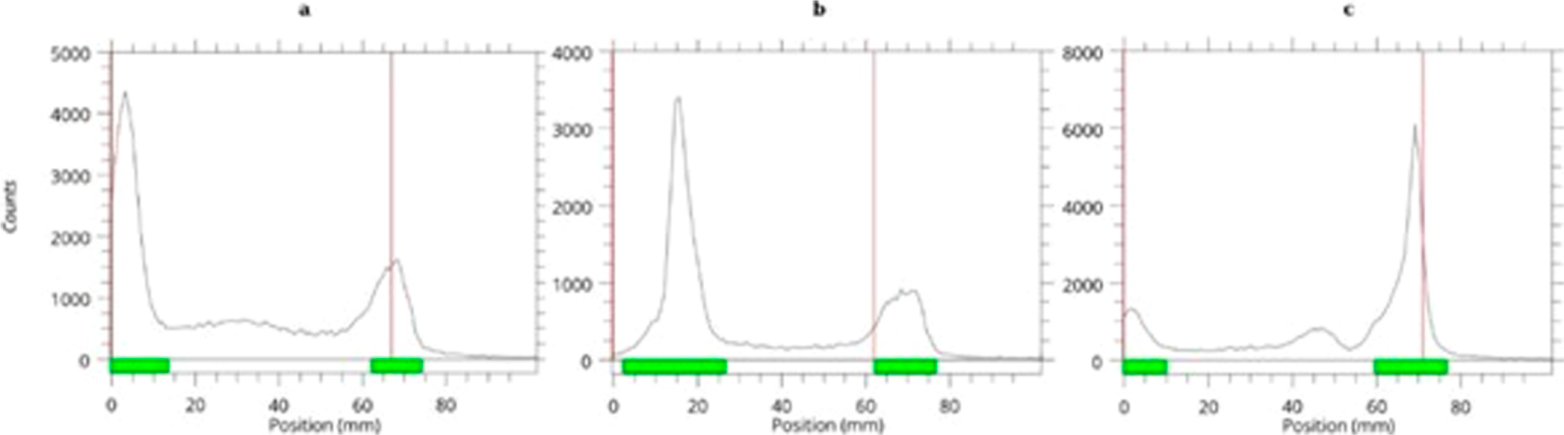
TLRC chromatograms of ^89^Zr-DFO-Fe_3_O_4_-mSi-NH_2_-Mn-Tra nanoparticles in (a) PBS, (b) DTPA, and (c) citric acid mobil phases.

**Figure 5:**
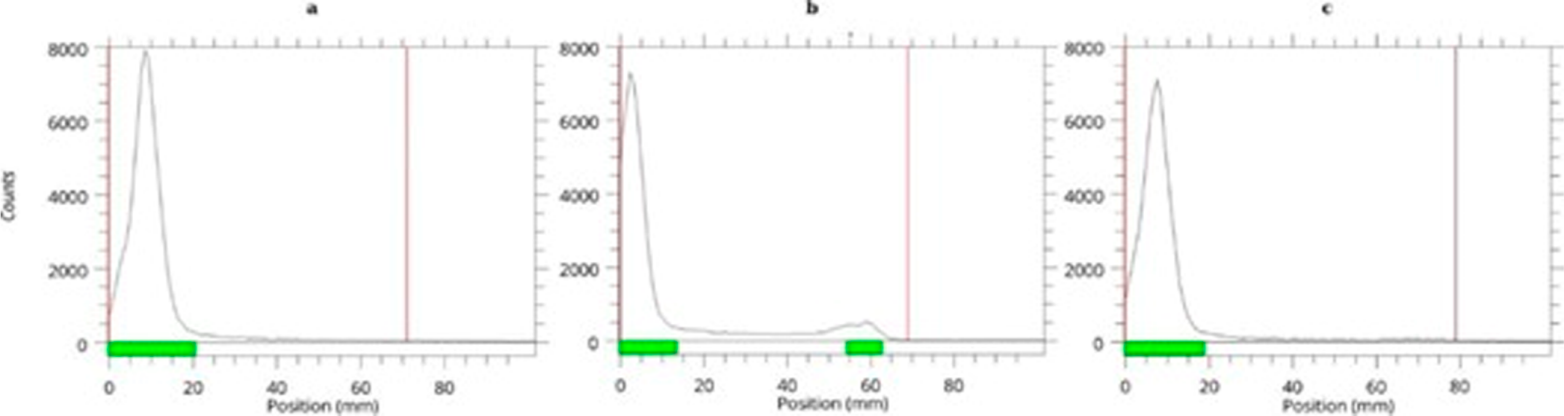
TLRC chromatograms of ^89^Zr-DFO-Tra in (a)­PBS, (b) DTPA, and (c) citric acid mobil phases.

**Table 3:** Radiolabeling Efficiency (%) as a Function of Incubation Time During Optimization

	Time (h)			
radiolabeled compounds	0.5	1	2	4
^89^Zr-DFO-Tra	98 ± 2	99 ± 1	88 ± 2	93 ± 3
^89^Zr-DFO-Fe_3_O_4_-mSi-NH_2_-Mn-Tra	78 ± 2	76 ± 1	69 ± 2	82 ± 1

### 3.3 Stability and Lipophilicity Results

Radiochemical stability of ^89^Zr-DFO-Fe_3_O_4_-mSi-NH_2_-Mn-Tra and ^89^Zr-DFO-Tra was evaluated up to 24 h to assess their suitability as PET radiopharmaceuticals. Both radioconjugates retained high radiochemical through incubation period (Figure 6). Degradation of ^89^Zr-DFO-Fe_3_O_4_-mSi-NH_2_-Mn-Tra, was only ∼8% at 24 h, a remaining radiochemical purity of approximately 92%. In comparison, ^89^Zr-DFO-Tra showed ∼2% degradation, maintaining ∼98% radiochemical purity at 24 h. The slightly lower stability observed for ^89^Zr-DFO-Fe_3_O_4_-mSi-NH_2_-Mn-Tra compared with ^89^Zr-DFO-Tra is might be due to the more complex surface environment of the nanoparticle and the potential for partial exposure of chelators at the particle–solvent interface. Nevertheless, the utilization of ^89^Zr-labeled nanomaterials for preclinical stability evaluation at 24 h is widely regarded as acceptable, so imaging was performed for a day administration. The high stability of ^89^Zr-DFO-Tra is consistent with previous reports, which demonstrated that DFO provides kinetically robust chelation of Zr^4+^ in aqueous media. This finding confirms that conjugation of the chelator to the nanoparticle platform does not eliminate this favorable behavior.^26,28^ Linear regression analysis revealed no statistically significant time-dependent degradation trend for either ^89^Zr-DFO-Fe_3_O_4_-mSi-NH_2_-Mn-Tra (slope = −0.145%/h, *R* ^2^ = 0.047, *p* = 0.438) or ^89^Zr-DFO-Tra (slope = −0.045%/h, *R* ^2^ = 0.002, *p* = 0.876), confirming the absence of systematic dissociation of ^89^Zr from the DFO chelate over the 24 h evaluation period. These data indicate that the ^89^Zr-DFO-Fe_3_O_4_-mSi-NH_2_-Mn-Tra construct maintains sufficient radiochemical stability over the evaluated period to support further preclinical PET-oriented investigations.

**Figure 6:**
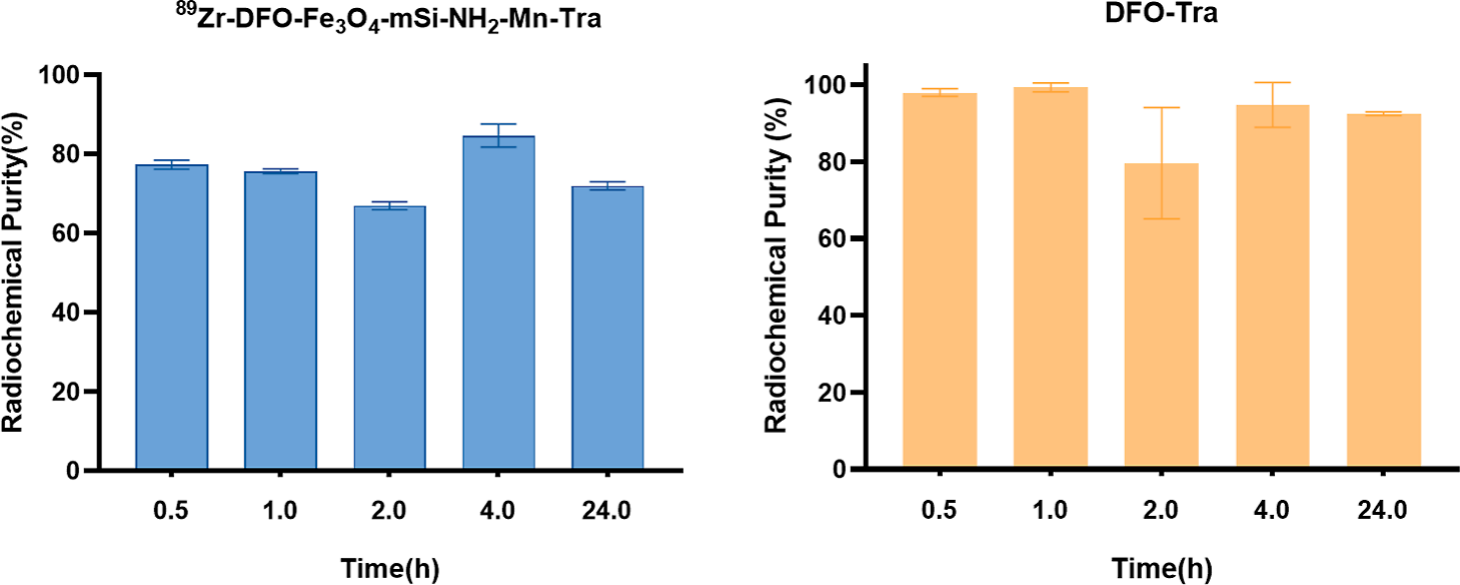
Radiochemical stability of ^89^Zr-DFO-Fe_3_O_4_-mSi-NH_2_-Mn-Tra (left) and ^89^Zr-DFO-Tra (right) in PBS (pH 7.4) at 37 °C over 24 h, assessed by TLRC. Data are expressed as mean ± SD (*n* = 3). Linear regression analysis revealed no significant time-dependent degradation trend for either formulation (NP: slope = −0.145%/h, *p* = 0.438; Tra: slope = −0.045%/h, *p* = 0.876).

Lipophilicity was evaluated by determining the *n*-octanol/water partition coefficient (log *P*), a key parameter that reflects a compound’s affinity for lipid versus aqueous phases and is known to influence membrane permeability and bioavailability. In general, compounds with log *P* values above 1 are considered sufficiently lipophilic to more readily interact with lipid bilayer membranes.^40^ The ^89^Zr-DFO-Fe_3_O_4_-mSi-NH_2_-Mn-Tra nanoparticle exhibited a log *P* of 2.23 ± 0.24, indicating lipophilicity. In contrast, log *P* of ^89^Zr-DFO-Tra showed to be 0.52 ± 0.15, consistent with a hydrophilic character and substantially lower partitioning into the lipid-like phase compared with the nanoparticle. These findings suggest that both constructs possess sufficient aqueous compatibility for systemic administration, while the ^89^Zr-DFO-Fe_3_O_4_-mSi-NH_2_–Mn-Tra nanoparticle displays a substantially higher lipophilic contribution than the antibody. The lipophilicity of the radiolabeled nanoparticle expected to facilitate its interaction with cellular membranes and in combination with receptor-specific binding mediated by the trastuzumab may contribute to the enhanced uptake observed in HER2-overexpressing cells.

### 3.4 In Vitro Cellular Uptake and Receptor Blocking

The receptor-mediated uptake of ^89^Zr-DFO-Fe_3_O_4_-mSi-NH_2_–Mn-Tra nanoparticles was evaluated in HER2-positive (SKBR-3) and HER2-negative (MDA-MB-231) breast cancer cell lines. In SKBR-3 cells, the uptake reached of 65.25 ± 3.2% at 2 h, while in MDA-MB-231 cells, the maximum uptake was 20.22 ± 1.8% at 4 h (Figure 7). Statistical analysis was performed by two-way ANOVA test (***p < 0.001 at each time point). Two-way ANOVA revealed a highly significant effect of cell line on uptake for both the nanoparticle *p* < 0.001, η^2^ = 0.919 and antibody (*p* < 0.001, η^2^ = 0.933) constructs, confirming that uptake was substantially and consistently higher in SKBR-3 cells than in MDA-MB-231 cells.

**Figure 7:**
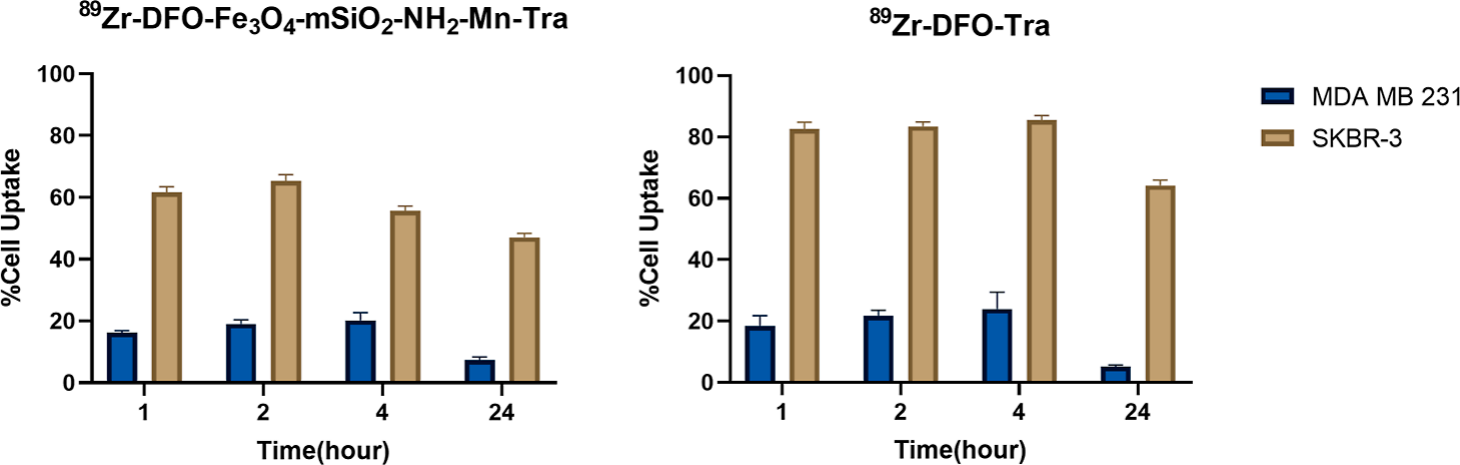
^89^Zr–Fe_3_O_4_-mSi-NH_2_-Mn-Tra radiolabeled nanoparticles and ^89^Zr-DFO-Tra radiolabeled antibodies uptake in target cells SKBR-3 and control group (MDA-MB-231) at 1, 2, 4, and 24 h. Data are expressed as mean ± SD (*n* = 3). Statistical analysis was performed by two-way ANOVA followed by multiple comparisons test (***p < 0.001 at each time point).

Preincubation with free trastuzumab reduced uptake to 11.14 ± 1.2%, confirming HER2 specific binding and receptor blockade. To confirm receptor specificity, a competition assay was performed by preincubating cells with excess unlabeled trastuzumab (500 nM) before addition of the radiolabeled construct. Two-way ANOVA with cell line and blocking condition as factors revealed a significant interaction effect (*p* < 0.001), indicating that trastuzumab pretreatment selectively affected uptake in a cell line-dependent manner. In HER2-positive SKBR-3 cells, receptor saturation significantly reduced ^89^Zr-DFO-Fe_3_O_4_-mSi-NH_2_-Mn-Tra uptake from 57.99 ± 2.31% to 46.85 ± 1.15% (*p* = 0.002), confirming HER2-mediated binding. In contrast, no significant change in uptake was observed in HER 2-negative MDA-MB-231 cells upon blocking (8.67 ± 2.0% vs 9.47 ± 1.0%; *p* = 0.569), validating the selectivity of trastuzumab-mediated targeting (Figure 8).

**Figure 8:**
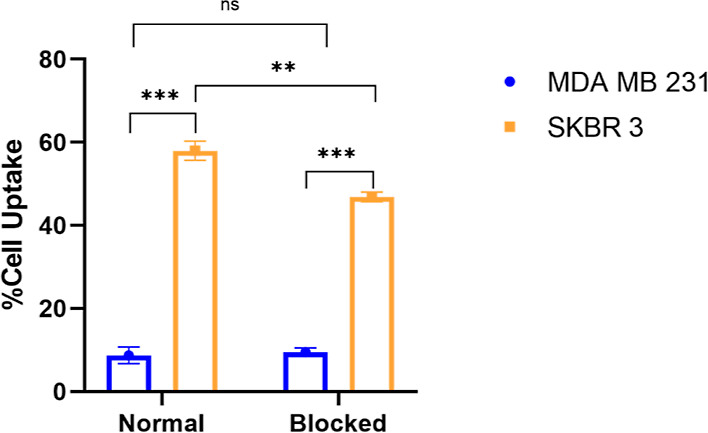
Uptake of ^89^Zr-DFO-Fe_3_O_4_-mSi-NH_2_-Mn-Tra in SKBR-3 (HER2^+^) and MDA-MB-231 (HER2^–^) cells with and without preincubation with excess unlabeled trastuzumab (500 nM). Data are expressed as mean ± SD (*n* = 3). Statistical analysis was performed by two-way ANOVA (factors: cell line and blocking condition) followed by multiple comparisons test ***p < 0.001, **p < 0.05; n.s., not significant.

In the preceding studies, the internalization of HER2-targeted ^89^Zr-labeled nanoparticles and antibodies in receptor-overexpressing cells was demonstrated to be via receptor-mediated endocytosis.^41–44^ The enhanced uptake in SKBR-3 cells is due to the high-affinity binding of trastuzumab’s Fab region to the extracellular domain IV of HER2, which promotes receptor clustering and subsequent internalization.^45^

The high uptake at 2 h suggests rapid receptor enter and endosomal accumulation. The difference between nanoparticle and antibody-only uptake rates reflects the larger hydrodynamic size and reduced diffusion coefficient of the nanoparticle conjugates compared with free antibodies.

These results confirm that surface-conjugated trastuzumab preserves biological activity postchelation and radiolabeling. The specific HER2 targeting is consistent with prior immuno-PET findings using ^89^Zr-DFO-Tra and highlights the potential of nanoparticle conjugation to advance multivalent receptor interactions and imaging signal-to-noise ratio.^32,46,47^

### 3.5 Biodistribution Results

The biodistribution profile of the ^89^Zr-DFO-Fe_3_O_4_-mSi-NH_2_-Mn-Tra nanoparticle, developed as a PET/MR candidate nanoplatform, was investigated in healthy rats to obtain baseline information on organ distribution and clearance. This type of biodistribution assessment is important because it demonstrates in which organs the nanoparticles accumulate, to what extent they remain in circulation, and whether they cross into the brain. This information indicates critical organs and helps predict where background signal might be high during imaging.

Organ-level distribution of ^89^Zr-DFO-Fe_3_O_4_-mSi-NH_2_-Mn-Tra nanoparticles was evaluated 1, 2, 4, and 24 h after injection (Figure 9). Organ uptake varied across the 1, 2, 4, and 24 h time points, with the highest liver uptake observed at 120 min (15.53 ± 7.6% ID/g) and particularly in the spleen. This result indicates that the nanoparticle tends to accumulate in these two organs in particular.^48^ This finding is important for safety assessment in repeated-use scenarios. Additionally, the high background signal in the liver and spleen may create a limitation that could reduce image contrast in regions near these areas. Kidney uptake increased at later time points. Blood uptake did not completely disappear over time. This suggests that part of the signal seen in some tissues in the early period may be influenced by the circulating fraction.

**Figure 9:**
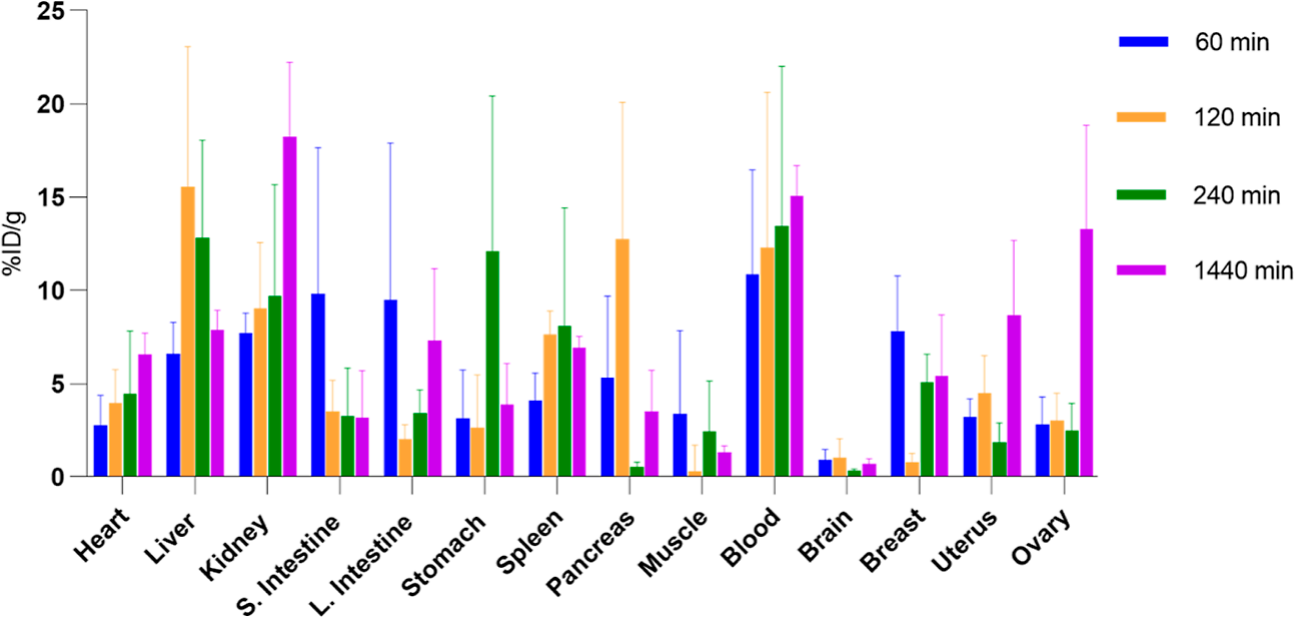
Biodistribution profiles of ^89^Zr-DFO-Fe_3_O_4_-mSi-NH_2_-Mn-Tra nanoparticles in healthy female Wistar rats at 60, 120, 240, and 1440 min postinjection. Data are expressed as mean % ID/g ± SD (*n* = 3 per time point). One-way ANOVA was performed for each organ across time points. Statistically significant time-dependent changes were observed in kidney breast, uterus (*p < 0.05) and ovary (**p = 0.006).

The very low brain activity at all time points is an important finding suggesting that the agent did not cross the BBB to a meaningful degree. This is advantageous in terms of reducing off-target brain exposure and facilitating interpretation in intracranial regions. In healthy animal data, low uptake was observed in breast, uterine, and ovarian tissues. Such biodistribution profiles are characteristic of silica-coated SPIONs, where hydrodynamic size, surface charge, and surface functionalization collectively determine their interactions with the mononuclear phagocyte system (MPS) and influence their circulation half-life. However, compared with unmodified SPIONs, the mesoporous silica coating and trastuzumab functionalization likely reduced nonspecific RES capture by introducing steric hindrance.^49^ The observed renal clearance pathway suggests gradual enzymatic or oxidative degradation of the silica shell, releasing small Mn/Fe complexes (<5 nm) that are filtered by the glomeruli. Similar partial renal elimination patterns have been reported for hybrid Mn–Fe_3_O_4_ systems.^16,50^ Consequently, the absence of brain or muscle accumulation indicates low off-target binding and a favorable safety profile for clinical translation.

However, the study was carried out healthy rats model, these breast, uterine, and ovarian findings are specifıcally, effective for HER2 targeting, altough they remain noteworthy. Although biodistribution studies in healthy animals showed relatively low uptake in breast, uterine, and ovarian tissues, both MR- and ^89^Zr-based PET in vitro studies demonstrated markedly increased accumulation in HER2-positive SKBR-3 cancer cells compared with HER2-negative controls. The findings suggest that, under pathological conditions characterized by HER2 overexpression, such as HER2-positive breast, uterine, or ovarian tumors, the nanoplatform may exhibit increased tumor-specific accumulation. The biodistribution data obtained in this study define the physiological distribution, background signal, and potential off-target accumulation sites of the PET/MR candidate agent and may serve as a basis for future tumor-targeting and tumor-contrast studies.

A major limitation of the present study is that direct in vivo PET or MRI imaging was not performed for the ^89^Zr-labeled construct. Although the parent nanoplatform was previously evaluated for MRI-related performance, those data were obtained only at the in vitro level. Therefore, the current findings support the potential of this system as a HER2-targeted PET/MRI candidate nanoplatform, but definitive validation of imaging performance and pharmacokinetics will require dedicated in vivo PET and MRI studies in HER2-positive tumor-bearing models.

## 4 Conclusion

The study demonstrates that ^89^Zr-DFO-Fe_3_O_4_-mSi-NH_2_-Mn-Tra can be obtained with high radiochemical efficiency and its physicochemical properties obtained for in vivo imaging suitable. Radiolabeling yields were 78 ± 2% for ^89^Zr-DFO-Fe_3_O_4_-mSi-NH_2_-Mn-Tra and 98 ± 2% for ^89^Zr-DFO-Tra. The nanoparticle formulation exhibited high stability in PBS and lipophilic (log *P* = 2.23 ± 0.24). Radiolabeled nanoparticles in SKBR-3 cells (HER2-positive) exhibited markedly higher uptake (65.25% for2 h) when compared with MDA-MB-231 cells (20.22% for 4 h). Receptor saturation reduced uptake by 11%, indicating HER2-mediated internalization. Biodistribution showed liver accumulation (15.53% ± 7.6 ID/g) and renal clearance with negligible brain uptake, indicating a RES-compatible and neuro-safe pharmacokinetic profile. When compared with analogous compounds documented in the extant literature, the construct demonstrates comparable or superior labeling stability, receptor binding and biodistribution.

This study extends our previous work by showing that the trastuzumab-functionalized manganese–iron oxide core–shell nanoparticle exhibits HER2-associated behavior in MRI-related in vitro assessment and in ^89^Zr-based PET-oriented in vitro/in vivo evaluation. In an earlier study, the nanoplatform exhibited selective intracellular accumulation in HER2-positive SKBR-3 cells, resulting in significant dual T_1_/T_2_ MR contrast enhancement. Meanwhile, minimal signal changes were observed in HER2-negative cells. Consistent with these observations, the present PET-focused investigation demonstrates higher, HER2-receptor-associated uptake of the ^89^Zr-labeled nanoparticle in SKBR-3 cells than in HER2-negative MDA-MB-231 cells. Taken together, the concordance between the previous MRI-related in vitro findings and the present ^89^Zr-based uptake data suggests that the developed nanoparticle merits further investigation as a dual-modality PET/MR candidate nanoplatform. Further preclinical optimization and translational studies might be required to evaluate the full potential of the ^89^Zr-DFO-Fe_3_O_4_-mSi-NH_2_-Mn-Tra nanoplatform for PET/MR imaging. Overall, these findings support ^89^Zr-DFO-functionalized manganese–iron oxide core–shell nanoparticles as a promising HER2-targeted PET/MRI candidate nanoplatform that warrants further validation by dedicated in vivo PET and MRI imaging studies.

## Supplementary Material



## Notes

Figure S1: DLS size distribution histograms and zeta potential profiles of Fe_3_O_4_-mSi-NH_2_-Mn-Tra and DFO-Fe_3_O_4_-mSi-NH_2_-Mn-Tra nanoparticles (PDF)
