# Supplementary material for: Evaluation of 89Zr-DFO Functionalized Manganese–Iron Oxide Core–Shell Nanoparticles as a HER2-Targeted Multimodal Imaging Candidate Nanoplatform
Source: ACS Omega. 2026 Jun 22;11(26):38684–94. doi: 10.1021/acsomega.6c01561 (PMC13347365; doi:10.1021/acsomega.6c01561)
Supplement: Supplementary file 1 [file ao6c01561_si_001.pdf]

SUPPORTING INFORMATION

Evaluation of ^{89}Zr-DFO Functionalized Manganese–Iron Oxide Core-Shell Nanoparticles as a HER2-Targeted Multimodal Imaging Candidate Nanoplatform

Derya Özel[1,2], Ayça Tunçel Oral[3], Selin Güleç[4], Fatma Yurt[1]*

[1]*Ege University, Institute of Nuclear Sciences, Department of Nuclear Applications, Bornova, Izmir, Türkiye*

[2]*Izmir University of Economics, Medical Imaging Techniques Program, Vocational School of Health Services, Izmir, Türkiye*

[3]*Cappadocia University, Medical Imaging Techniques Program, Cappadocia Vocational College, Health Campus, 50420, Nevsehir, Türkiye*

[4]*Ege University, The Institute of Natural and Applied Sciences, Department of Biotechnology, 35100, Izmir, Türkiye*

Hydrodynamic Size and Zeta Potential Analysis of DFO-Conjugated Nanoparticles

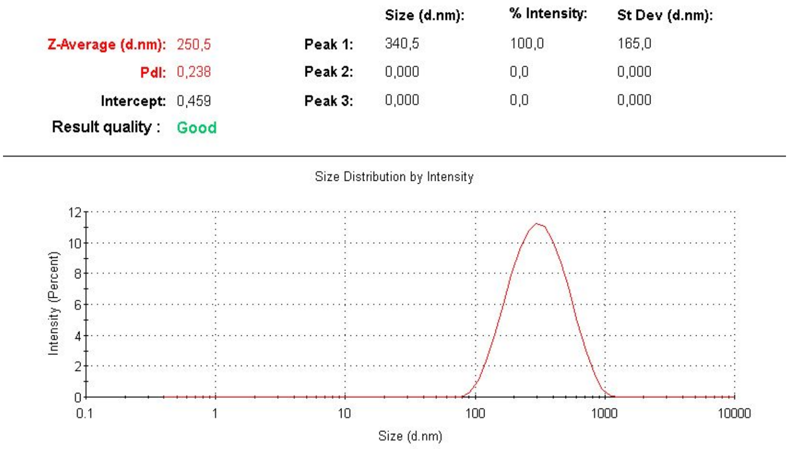

Figure S1 a).Fe_3O_4-mSi-NH_2-Mn-Tra nanoparticles DLS size results

			Size (d.nm):	% Intensity:	St Dev (d.nm):
Z-Average (d.nm):	231,8	Peak 1:	262,0	100,0	90,34
PdI:	0,057	Peak 2:	0,000	0,0	0,000
Intercept:	0,490	Peak 3:	0,000	0,0	0,000
Result quality :	Good				

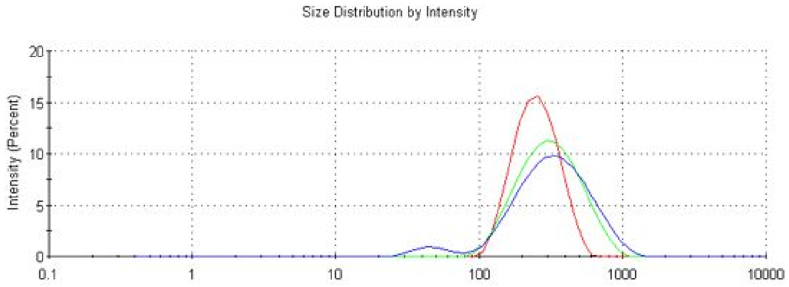

Figure S1b) Fe_3O_4-mSi-NH_2-Mn-Tra nanoparticles DLS size triple results

			Mean (mV)	Area (%)	St Dev (mV)
Zeta Potential (mV):	-18,5	Peak 1:	-11,3	55,9	4,78
Zeta Deviation (mV):	10,2	Peak 2:	-27,6	44,1	6,30
Conductivity (mS/cm):	1,44	Peak 3:	0,00	0,0	0,00
Result quality :	Good				

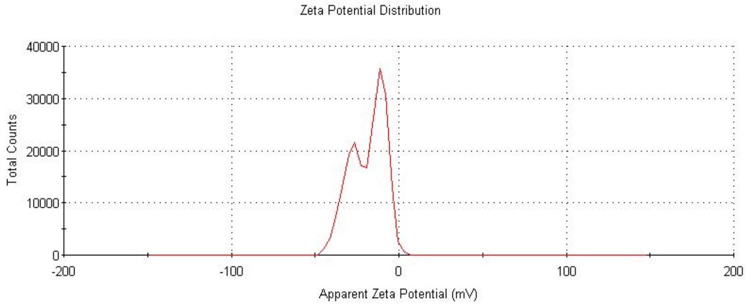

Figure S1 c) Fe_3O_4-mSi-NH_2-Mn-Tra nanoparticles Zeta Potential results

			Size (d.nm):	% Intensity:	St Dev (d.nm):
Z-Average (d.nm):	294,2	Peak 1:	417,5	100,0	277,5
PdI:	0,292	Peak 2:	0,000	0,0	0,000
Intercept:	0,307	Peak 3:	0,000	0,0	0,000
Result quality :	Good				

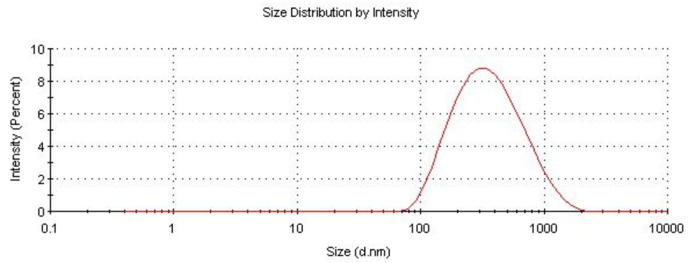

Figure S1 d) DFO-Fe_3O_4-mSi-NH_2-Mn-Tra nanoparticles DLS size results

			Size (d.nm):	% Intensity:	St Dev (d.nm):
Z-Average (d.nm):	294,9	Peak 1:	309,1	92,8	158,7
PdI:	0,442	Peak 2:	5048	7,2	583,8
Intercept:	0,845	Peak 3:	0,000	0,0	0,000
Result quality :	Good				

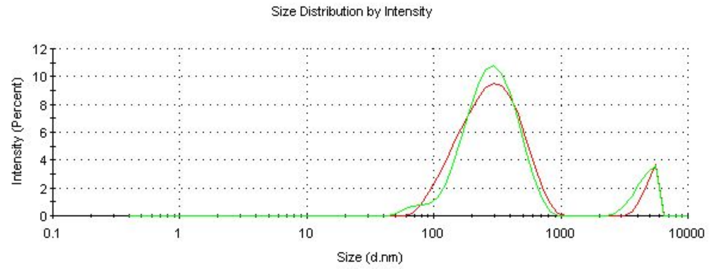

Figure S1 e) DFO-Fe_3O_4-mSi-NH_2-Mn-Tra nanoparticles DLS size triple results

			Mean (mV)	Area (%)	St Dev (mV)
Zeta Potential (mV):	-16,2	Peak 1:	-16,2	100,0	5,95
Zeta Deviation (mV):	5,95	Peak 2:	0,00	0,0	0,00
onductivity (mS/cm):	0,00555	Peak 3:	0,00	0,0	0,00
Result quality :	Good				

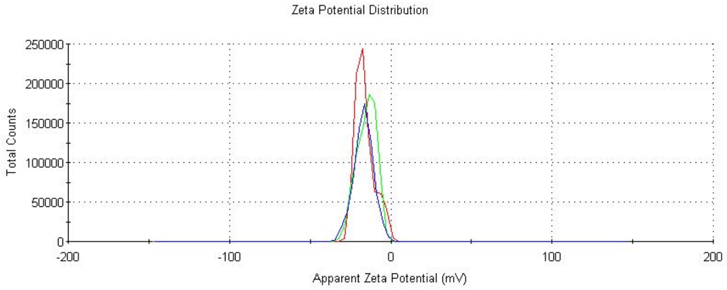

Figure S1 f) DFO-Fe_3O_4-mSi-NH_2-Mn-Tra nanoparticles DLS zeta triple results
